# A health sciences researcher’s experience of manuscript review comments, 2020–2022

**DOI:** 10.4102/safp.v65i1.5753

**Published:** 2023-10-25

**Authors:** Gina Joubert

**Affiliations:** 1 Department of Biostatistics, Faculty of Health Sciences, University of the Free State, Bloemfontein, South Africa

**Keywords:** review comments, manuscript review, publication, peer review, review feedback, experience, health sciences

## Abstract

**Background:**

Peer review frequently improves a manuscript, but authors may consider some reviewer feedback negative, inappropriate or unclear. This study aims to summarise and analyse review comments received by authors.

**Methods:**

This longitudinal study included all submissions of which the researcher was an author, reviewed by any journal during 2020–2022. First-round reviews were retrieved from emails and documents received by the authors or the faculty’s medical editors or the journal platforms. A confidential datasheet with review items compiled from literature and the researcher’s experience as author and reviewer was completed for each submission. Review comments were noted verbatim for subjective items such as rude or vague statements.

**Results:**

The 65 submissions received 118 reviews from 36 journals, mainly in the form of unstructured narrative reports (59%). The majority of first-round reviews (58%), including those for rejected submissions, contained some positive comments. Reviewers frequently (75% of reviews, 88% of submissions) required some expansion of information. Vague and incorrect statements occurred in 15% and 18% of reviews, respectively. Only two reviews contained statements that could be considered rude. The types of comments made were associated with the review format.

**Conclusion:**

The majority of reviews contained some positive comments and rude comments were extremely rare. Reviewers frequently requested the expansion of information provided.

**Contribution:**

This study gives insight to authors, reviewers and editors regarding the type and tone of review comments. This could guide authors during manuscript preparation and authors, reviewers and editors during the review process.

## Introduction

Peer review is the cornerstone of scientific research publication. The review process frequently improves a manuscript,^1^ and there is a call for reviewing to be acknowledged and rewarded as a scholarly activity.^2,3^ Authors, however, also receive reviewer feedback that they consider ‘ill-informed or biased’.^4^

Datta^5^ provided a list of points to ‘help researchers and scientists to focus their energy to improve the quality of peer reviews’. Although most are important issues for authors and editors, an author is unaware whether a specific reviewer fulfilled the points related to reviewer timeliness, continuity and confidentiality. But authors can judge whether a reviewer is courteous, constructive or has mistaken the review role as that of being the copy editor. The Committee on Publication Ethics (COPE) has an overarching guideline focusing on the ethical aspects of reviewing.^6^ Journals or journal publishers usually provide their guidelines, frequently based on the COPE guidelines.

### Aim

This study aimed to summarise and analyse manuscript review comments received during 2020–2022. As researchers may focus on particularly negative or poor reviews, an analysis of review feedback could give perspective on the type of comments received. Associations between review format and types of feedback received were also investigated.

## Research methods and design

### Study design and sampling

In this historical longitudinal study, all manuscripts of which G.J. is an author, reviewed by any journal during 2020–2022, were included. Manuscripts reviewed during this period by more than one journal because of a rejection by the first journal were considered separate submissions. Manuscripts rejected or accepted during the study period without being sent for review were excluded.

### Data collection

The researcher retrieved reviews of the first review round from emails she had received as a co-author or requested emails from corresponding authors or the medical editors of the Faculty of Health Sciences of the University of the Free State (UFS) or from the journal platforms. A confidential review datasheet was completed for each submission. This datasheet included items identified in the literature and from the researcher’s experience as author and reviewer. Only items that could be completed using the review and the submitted manuscript were included. For subjective items, such as whether rude terms were used or reviewers made vague statements, comments were noted verbatim. Comments were considered vague if statements indicated that changes or expansion of information were required, but no indication was given of what the change or expansion should consist of. A comment such as ‘The Conclusion needs to be redone’ (with no details provided) was considered vague. Comments were considered incorrect if methodological terminology was requested that was inappropriate for the study approach, analyses were requested that were not appropriate for the study aim or the type of data available or if comments contradicted the journal’s requirements or practice. Comments containing words such as *excellent, good, well done* were considered positive. Data were collected during the first half of 2022 (reviews: 2020–2021), September 2022 (reviews: January 2022 – June 2022) and March 2023 (reviews: July 2022 – December 2022).

### Pilot study

A pilot study to test the datasheet and the accessing of reviews was done on the first four reviewed submissions of 2020 (eight reviews). Subsequently, additional items and answer options were added to the datasheet. The pilot study cases were included in the main study after the revised datasheets were completed.

### Ethical considerations

The protocol, amended datasheet and amendments to extend the study period were approved by the Health Sciences Research Ethics Committee of the UFS (UFS-HSD2022/0001/2202). No reviewers were identified as their identities are unknown to the author. Authors of papers (other than the author) were not identified. To maintain confidentiality, manuscripts were identified by consecutive numbers on the datasheet, with a separate list linking these numbers to manuscript titles.

### Statistical analysis

The researcher analysed the data using SAS version 9.4, as frequencies and percentages. Associations between categorical variables were investigated using chi-squared or Fisher’s exact tests.

## Results

During the study period, 67 submissions were reviewed. Reviewer comments could be located for 65 submissions (97%). For 63 submissions, complete review documentation was available for data collection; for two, only author responses to reviewer comments were available.

Table 1a and Table 1b summarises submission, review and outcome characteristics. Submissions were made to 36 journals, of which 23 were South African-based. Nearly 60% of reviews contained some positive comments, and 74% of submissions received some positive comments. For rejected submissions, this percentage was 56% (10 of 18 submissions). Reviewers frequently (75% of reviews, 88% of submissions) required some expansion of information (without commenting on how this would impact word counts). Incomprehensible comments comprised mainly garbled typing in comment boxes or poor punctuation or phrasing, making understanding difficult or impossible. Comments indicating that the reviewer had misunderstood the information provided or had not read the manuscript closely consisted mainly of the reviewer indicating that details were missing, whereas the details were stated in the manuscript.

**TABLE 1a:** Submission, main review and outcome characteristics (*n* = 65).

Variable	*n*	%
**Manuscript type**		
Full-length article	62	95
Short report	2	3
Letter to editor	1	2
**Study field**		
Clinical medicine	39	60
Laboratory	5	8
Allied Health Professions	7	11
Education	11	17
Other	3	5
**Research type**		
Undergraduate student project	19	29
Master of Medicine project	23	35
Other postgraduate project	17	26
Staff project	5	8
Other	1	2
**Study type**		
Descriptive	8	12
Cross-sectional	32	49
Comparative	2	3
Cohort analytical	20	31
Randomised controlled trial	1	2
Scoping review	1	2
Delphi	1	2
**Data collection**		
Prospective (research data)	38	58
Retrospective (existing data)	24	37
Both	3	5
**Journal base**		
South Africa	51	75
Africa	2	3
Elsewhere (international)	12	18
**Number of reviewers (*n* = 61)**		
One	13	21
Two	44	72
Three	3	5
Four	1	2
**Outcome of first round of review**		
Revisions required	49	75
Rejected	16	25
**Final outcome of review process**		
Accepted	45	69
Rejected	18	28
Withdrawn by author	1	2
Archived by journal because of late response by authors	1	2

**TABLE 1b:** Submission, main review and outcome comments.

Type of comments	Per submission (*n* = 65)		Per review (*n* = 118)	
	*n*	%	*n*	%
Any positive comment	48	74	68	58
Any expansion of information required	57	88	89	75
Any vague comments	15	23	18	15
Any incomprehensible comments	10	15	10	8
Any wrong and/or inappropriate comments	19	29	21	18
Any comments indicating the reviewer has not understood the information provided or has not read the manuscript closely	14	22	15	13
Any rude, derogatory and/or destructive comments	2	3	2	2
Any comments that imply change(s) to the ethics committee-approved protocol	16	25	20	17
Any comments that indicated that the entire study has to be redone	8	12	8	7
Reviewer did copy-editing	12	18	13	11

Two reviews contained *possibly rude, derogatory and/or destructive* comments:

- ‘*the referencing is awful*’ and numerous exclamation marks by the reviewer after other statements.
- ‘*The scientific rigour of this paper is very weak. I am not convinced that the authors understood the method they used*’.

Comments that implied changes to the ethics committee-approved protocol comprised mainly requests for additional types of data to be collected or data to be collected in a different way. Copy-editing occurred frequently. Five reviews (4%) comprised of only language editing.

Reviews were done in a variety and combination of formats, the most common being an unstructured narrative report (59%). Forty-seven percent (*n* = 54) comprised only an unstructured narrative report, 19% (*n* = 23) only a report structured by the reviewer and 10% (*n* = 11) only comments and/or track changes in the manuscript. Twenty-four reviews (20%) contained answers to items and/or questions specified by the journal.

Significant associations were found between review format and types of reviewer comments:

- Reviews consisting only of track changes and/or comments in the manuscript itself were least likely to have positive comments (36%, 4/11); most reviews (88%, 21/24) that contained responses to items and/or questions specified by the journal had positive comments (*p* = 0.01).
- Reviews consisting only of a report structured by the reviewer were most likely to have vague comments (30%, 7/23); reviews consisting only of a narrative report (7%, 4/54) the least likely (*p* = 0.05).
- Reviews consisting only of track changes or comments in the manuscript were more likely to contain copy-editing (45%, 5/11) than all other review formats (< 15%) (*p* = 0.01).

For 10 (15%) submissions, the editor or associate editor added their own comments to the reviewers’ feedback or was the sole reviewer of the manuscript. For 13 (20%) submissions, the editor or associate editor gave input at some point during the review process.

## Discussion

Reviewer comments were received in a wide range of formats. As highlighted by researchers in the surgical field, there is little uniformity regarding the review requirements stipulated by journals.^7^ To ensure that reviews are thorough and cover all aspects of the manuscript, some structured format seems preferable. However, reviewers do not necessarily give feedback in the format required by a journal. As editors of Educational Studies in Mathematics, Mesa et al.^8^ conceded that few reviewers answered the questions stipulated by their journal, and therefore, they had decided that for their journal, no specific review questions or criteria would be stipulated in future.

It was encouraging that most reviews (even those leading to rejection) contained positive feedback. This confirmed why such a structured investigation was necessary; otherwise, authors may remember only extreme or odd cases. As reviewers, it is important that a review should assess not only the weaknesses but also the strengths of a manuscript.^7^

Journals’ review instructions regarding copy-editing differ widely with some stating explicitly that no comment is needed regarding spelling, grammar and layout^9^ while others state that all spelling, grammar and typographical errors must be pointed out at the review stage as no further copy-editing is done later.^10^ COPE guidelines^7^ state that a reviewer should ‘not attempt to rewrite it to your own preferred style if it is basically sound and clear’.

In a review of 1491 sets of reviewer comments of manuscripts in the fields of Ecology and Evolution and Behavioural Medicine, Gerwing et al.^11^ found that 12% contained at least one unprofessional comment towards the author or their work. The two potentially rude comments identified in the current study would be considered unprofessional according to the definitions of Gerwing et al.^11^ Gerwing et al.^12^ report on the social media debate after their 2020 publication and state that the debate indicated that there is a negative cultural zeitgeist in peer review. Reviewers need to ‘put more effort into considering the perspective of authors when wording their comments’.^12^ In the current study, there was little evidence of such negativity. Open review, a developing trend in scientific publication,^13^ may also influence the tone and type of reviewer comments. Only one of the journals included in this study made use of transparent open review.

As pointed out by Roediger^14^ and Eva,^15^ reviewers, as readers of a submission, may misinterpret or misunderstand information provided. As reviewers frequently scrutinise submissions more closely than the usual reader, authors need to acknowledge that readers of the published manuscript may experience the same difficulties. Authors should regard such comments as flagging sections that need to be written more clearly.

Gerwing et al.^11^ reported that 41% of reviews contained incomplete, inaccurate or unsubstantiated comments. Generally, if authors can motivate in their response why they consider comments inappropriate or incorrect, the motivation will be accepted by the reviewers and/or editor.

Feedback implying changes to the ethics committee-approved protocol places the researcher in an ethical dilemma. The revision time allowed by journals is often 2–4 weeks, whereas approval by the ethics committee of a protocol amendment and thereafter completing the required new work (data collection, analysis) may require extensive time.

The American Speech Language Hearing Association describes the role of the editor to include monitoring the peer-review process ‘to ensure fairness, timeliness, thoroughness, and civility’.^16^ Tennant and Ross-Hellauer^17^ highlighted that the editorial role is, unfortunately, seemingly focused on decision-making rather than on the process leading to that decision. In a survey of editors’ opinions on peer review,^18^ most editors responded that a reviewer’s comments may be edited without his/her permission when a reviewer has used inappropriate or offensive language (58%).

Arthur^19^ has called for editors to become more active in pre-screening manuscripts, particularly in terms of language, scope, format and scientific quality. This would lessen the load on reviewers. The review pool is often limited or overburdened^2^ or, as has been shown through mathematical modelling, only ‘a small portion of the scientific community is carrying a disproportionate load of the peer review’.^20^

### Limitations and strength

A limitation of this study is that the occurrence of different types of comments is reported, not the frequency at which they occur per review. The study by Gerwing et al.^11^ followed the same approach. The addition of a co-investigator to independently complete the datasheet would have enhanced the validity of the data.

A strength of this study is the researcher’s intimate knowledge of the submitted manuscripts, an aspect that limited the type of information other researchers could investigate as they were not involved in the studies reviewed. The fact that data collection occurred at three different time points contributed to minimising researcher recall bias.

## Conclusion

Despite its small size and particular profile of manuscripts reviewed, this study gives some insight into what authors can expect from the review process in terms of type and tone of comments. These findings can guide authors during manuscript preparation and authors, reviewers and editors during the review process.

### Recommendations

- Journal editors need to be aware of the extent to which reviewers require expansion of information and clarify for authors and reviewers how this impacts prescribed word count limits.
- Journal editors should carefully consider the value of various review formats.
- Authors need to be aware of their right to counter or query reviewer comments, especially vague, incorrect or inappropriate comments.
- Ethics committees can deliberate on how best to expedite protocol amendments based on reviewer comments.
